# The Slow-Releasing Hydrogen Sulfide Donor, GYY4137, Exhibits Novel Anti-Cancer Effects *In Vitro* and *In Vivo*

**DOI:** 10.1371/journal.pone.0021077

**Published:** 2011-06-20

**Authors:** Zheng Wei Lee, Jianbiao Zhou, Chien-Shing Chen, Yujun Zhao, Choon-Hong Tan, Ling Li, Philip Keith Moore, Lih-Wen Deng

**Affiliations:** 1 Department of Biochemistry, National University of Singapore, Singapore, Singapore; 2 Department of Chemistry, National University of Singapore, Singapore, Singapore; 3 Department of Pharmacology, National University of Singapore, Singapore, Singapore; 4 Cancer Science Institute of Singapore, National University of Singapore, Singapore, Singapore; 5 Department of Medicine, National University of Singapore, Singapore, Singapore; 6 Division of Hematology and Oncology, School of Medicine, Loma Linda University, Loma Linda, California, United States of America; Bauer Research Foundation, United States of America

## Abstract

The slow-releasing hydrogen sulfide (H_2_S) donor, GYY4137, caused concentration-dependent killing of seven different human cancer cell lines (HeLa, HCT-116, Hep G2, HL-60, MCF-7, MV4-11 and U2OS) but did not affect survival of normal human lung fibroblasts (IMR90, WI-38) as determined by trypan blue exclusion. Sodium hydrosulfide (NaHS) was less potent and not active in all cell lines. A structural analogue of GYY4137 (ZYJ1122) lacking sulfur and thence not able to release H_2_S was inactive. Similar results were obtained using a clonogenic assay. Incubation of GYY4137 (400 µM) in culture medium led to the generation of low (<20 µM) concentrations of H_2_S sustained over 7 days. In contrast, incubation of NaHS (400 µM) in the same way led to much higher (up to 400 µM) concentrations of H_2_S which persisted for only 1 hour. Mechanistic studies revealed that GYY4137 (400 µM) incubated for 5 days with MCF-7 but not IMR90 cells caused the generation of cleaved PARP and cleaved caspase 9, indicative of a pro-apoptotic effect. GYY4137 (but not ZYJ1122) also caused partial G_2_/M arrest of these cells. Mice xenograft studies using HL-60 and MV4-11 cells showed that GYY4137 (100–300 mg/kg/day for 14 days) significantly reduced tumor growth. We conclude that GYY4137 exhibits anti-cancer activity by releasing H_2_S over a period of days. We also propose that a combination of apoptosis and cell cycle arrest contributes to this effect and that H_2_S donors should be investigated further as potential anti-cancer agents.

## Introduction

Hydrogen sulfide (H_2_S) is synthesized naturally from cysteine by several enzymes including cystathionine γ lyase (CSE), cystathionine β synthetase (CBS) and 3-mercaptosulfurtransferase (3-MST) in a wide range of mammalian and non-mammalian cells both *in vitro* and *in vivo*. In the last decade, numerous physiological and pathophysiological roles have been proposed for this gas along with a plethora of cellular and molecular targets including a range of ion channels, enzymes and transcription factors [1].

Apart from its potential roles in normal physiology there is also a comprehensive literature describing the toxicity of H_2_S and its role as an environmental pollutant [2]. A number of studies have investigated the role of H_2_S in triggering cell death and evidence has been presented that this gas can exert both pro- and anti-apoptotic activity in cultured cells [3], [4]. However, the precise mechanism(s) involved remain unclear.

Perhaps surprisingly, there have been few studies of the effect of H_2_S on cancer cells *in vitro* and no reports of its effect on tumor progression *in vivo*. Several years ago we reported that H_2_S protected colon cancer cells (HCT-116) from apoptosis due to β-phenylethyl isothiocyanate [5]. Others have subsequently reported that H_2_S increases human colon cancer cell proliferation and reduces apoptosis in several cell lines (e.g. HCT-116, [6]) whilst decreasing survival in other human colon cell lines (e.g. WiDR, [7]). These disparate observations are difficult to reconcile. However, one explanation may lie in the choice of H_2_S donor. Sulfide salts such as sodium hydrosulfide (NaHS) and sodium sulfide (Na_2_S) have been widely used to study the biological effects of this gas in many cells, tissues and animals. On addition of water, these salts generate a large amount of H_2_S over a short time period. Since cell culture takes place over a period of hours or days, it is likely that little, if any, H_2_S is present in medium within a short time of adding either NaHS or Na_2_S. Thus, whilst no direct measurements have been made up to this point, it seems likely that the concentration of H_2_S that cancer (or indeed other) non-cancer cells are exposed to during culture with NaHS will be high at the start and not sustained throughout the experiment. Thus, it may be difficult to draw firm conclusions about the ability of H_2_S to affect cancer cell survival using sulfide salts as donor agents.

With this in mind, we previously reported that GYY4137 releases H_2_S slowly both in aqueous media and when administered to intact animals over a period of hours to days [8], [9]. We have now compared the effect of GYY4137 and NaHS on survival of a range of cancer and non-cancer cells in culture and correlated their effect with changes in concentration of H_2_S in the medium. In addition, we have examined the effect of GYY4137 on tumor growth using a xenograft model in immunodeficient mice.

## Materials And Methods

Protocols were conducted with the approval of National University of Singapore (NUS) Institutional Review Board (IRB, reference code: 09-120E) and NUS Institutional Animal Care and Use Committee (IACUC, protocol number: 804/05).

### Chemical synthesis of GYY4137 and ZYJ1122

GYY4137 was synthesized chemically in house as described previously [8]. ZYJ1122 (morpholin-4-ium diphenylphosphinate) was synthesized as follows. To a solution of diphenylphosphinic acid (1.0 mmol, 1.0 equiv.) in dichloromethane (DCM; 2 ml) at room temperature, morpholine (2.0 mmol, 2.0 equiv.) was added drop-wise. The reaction was stirred at the same temperature for 1 hour and the product subsequently collected by suction filtration. The pure product was obtained after washing with cold DCM. White solid was obtained as 56% yield. ^1^H NMR (500 MHz, CDCl_3_, ppm): δ  = 7.77–7.74 (m, 4H), 7.38–7.32 (m, 6H), 3.77–3.75(m, 4H), 2.95–2.93(m, 4H); LRMS (ESI) m/z 217.2 (M^−^). The purity and structures of the compounds were verified using Proton Nuclear Magnetic Resonance Spectrometry (^1^H NMR) and Mass Spectrometry (Figures S1, S2, S3, S4).

### Measurement of H_2_S

The generation of H_2_S from either NaHS (Sigma), GYY4137 or ZYJ1122 (all 400 µM) was determined in aliquots (100 µl) withdrawn at timed intervals (up to 7 days) from cultured MCF-7 cells maintained in Dulbecco's modified Eagle's medium (DMEM; Sigma) as described below. The concentration of H_2_S (determined as a combination of free H_2_S, HS^−^ and S^2−^) was measured spectrophotometrically as described previously [10]. Briefly, medium (100 µl) was mixed with 0.85% w/v zinc acetate/3% NaOH mixture (1:1 ratio, 100 µl). Methylene blue was then formed by the addition of N,N-dimethyl-p-phenylenediamine-dihydrochloride dye and FeCl_3_ (final concentrations, 2.5 mM and 3.3 mM respectively) and absorbance subsequently monitored at 670 nm. The concentration of H_2_S (defined as above) was determined using a standard curve of NaHS (0–400 µM; R^2^ = 0.9987).

### Cell culture and cell viability

All the cell lines except HCT-116 were obtained from American Type Culture Collection (ATCC). Human cervical carcinoma (HeLa), colorectal carcinoma (HCT-116), hepatocellular carcinoma (Hep G2), osteosarcoma (U2OS), breast adenocarcinoma (MCF-7) and human diploid lung fibroblasts (IMR90 and WI-38) were cultured in DMEM supplemented with 10% v/v fetal bovine serum (FBS; HyClone), penicillin/streptomycin (100 U/ml; Sigma) and L-glutamine (2 mM; Caisson) at 37°C in an atmosphere of 5% CO_2_. Human acute promyelocytic leukemia cells (HL-60) were cultured in DMEM containing 20% v/v FBS whilst human myelomonocytic leukemia (MV4-11) cells were cultured in RPMI with 10% v/v FBS under the same incubation conditions. Live cell populations incubated with NaHS, GYY4137 or ZYJ1122 (400 or 800 µM) were collected after 5 days and counted in triplicate after staining with trypan blue using a haemocytometer. Concentration response for GYY4137 (100–1000 µM) were generated in MCF-7, HL-60 and MV4-11 exposed to drugs for 5 days and the ability to reduce survival assessed as IC_50_ values. Colony formation using MCF-7 cells was also assessed by a clonogenic survival assay as described elsewhere [11]. Briefly, MCF-7 cells (10,000) were seeded in triplicate in 6-well plates in the presence of GYY4137, NaHS or ZYJ1122 (200 to 600 µM) for 10 days until colonies were readily visible. Colonies were then stained with crystal violet (5% w/v) and the representative pictures were captured using a ChemiGenius 2 Bio Imaging System (SynGene Ltd).

### *In vivo* efficacy of GYY4137

The animal experimental protocol has been described previously [12]. Briefly, female, severe combined immunodeficiency (SCID) mice (17–20 g, 4–6 weeks old) were bred in house and maintained throughout in specific pathogen-free (SPF) isolators. Exponentially growing HL-60 and MV4-11 cells (1×10^7^) (>95% viability) were washed twice in phosphate-buffered saline and subcutaneously injected into the loose skin between the shoulder blades and the left front leg of recipient mice. Animals were treated with either GYY4137 (100, 200 and 300 mg/kg/day, i.p.) or saline (1 ml/kg/day, i.p.) for 14 days commencing 14 days after injection of cells at which point mice had palpable tumors of 100 mm^3^ average size All animals were closely monitored. Weight and tumor size were measured at daily intervals. For tumor size measurement, length (L) and width (W) of the tumor were measured with a caliper, and tumor volume (TV) was calculated as TV  =  (L×W^2^)/2.

### Cell Cycle Analysis and Western Blotting

MCF-7 cells (40,000) were incubated in 6-well plates in the presence or absence of GYY4137 (400 µM) for either 5 or 8 days. Cells treated with ZYJ1122 were used as a control. To analyze the cell cycle profile, cells were fixed with 70% v/v ethanol on ice for at least 2 hours and then stained in propidium iodide solution (20 µg/ml propidium iodide, 100 µg/ml RNase A and 0.1% v/v Triton X-100) for 15 min at 37°C. Stained cells were then subject to DNA content analysis by flow cytometry (Dako CyAn ADP) and the data obtained was processed using Summit software (Beckman Coulter). Cell lysates of MCF-7 were subjected to SDS-PAGE and transferred to polyvinylidene difluoride (PVDF) membranes. The membranes were blocked in Tris buffered saline (TBS) containing non-fat dry milk (5% w/v) and thereafter incubated with the relevant primary antibody (1 µg/ml) at 4°C overnight. Antibodies used were α-PARP, α-cleaved-PARP, α-cleaved-caspase 9 (Cell Signaling Ltd.) and α-tubulin (Sigma).

### Statistical analysis

Cell survival, IC_50_ and tumor volumes were expressed as mean ± standard error (SEM). For *in vitro* studies, cell survival of both non-treatment (NT) and treatment groups was analyzed using one-way ANOVA followed by a post-hoc t test. For *in vivo* studies, the comparisons between vehicle control group and different dosage treatments was analyzed using linear mixed model for longitudinal data analysis by SPSS software (IBM). *P*<0.05 was considered significant.

## Results

### Release of H_2_S from NaHS and GYY4137 in culture medium

**Figure 1:**
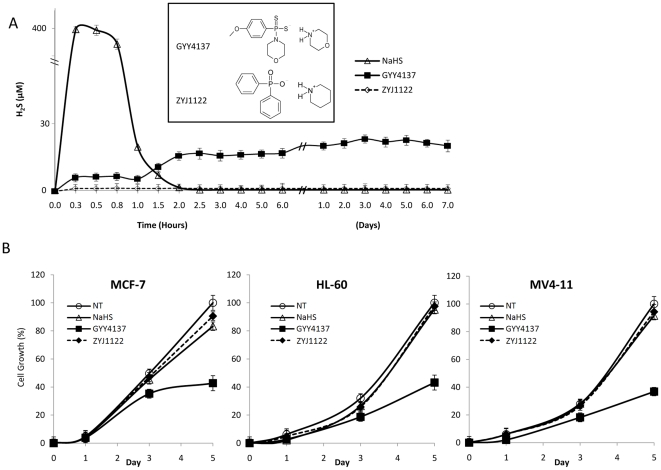
Differential H_2_S-releasing manner of NaHS and GYY4137. (A) H_2_S-releasing profile of NaHS, GYY4137 and ZYJ1122. H_2_S released from NaHS, and GYY4137 (400 µM) was determined in aliquots (100 µl) of medium withdrawn at timed intervals (up to 7 days) from cultured MCF-7 cells. Concentration of H_2_S amounts was assessed spectrophotometrically using N,N-dimethyl-p-phenylenediamine-dihydrochloride and results showed the H_2_S concentration (µM). H_2_S release from GYY4137 was significantly different (*P*<0.05) from T = 0 at all time points from 0.3 hour to 7 days. H_2_S release from NaHS was significantly different (*P*<0.05) from T = 0 at all time points up to 1.5 hours. No detectable H_2_S was released from ZYJ1122 (400 µM) under identical experimental conditons. Results show mean ± s.e. mean, n = 3. The chemical structures of GYY4137 and ZYJ1122 are shown in the inset. (B) Growth curve analyses of MCF-7, HL-60 and MV4-11 cells treated with NaHS, GYY4137 and ZYJ1122 (400 µM) over 5 days. Cell survival was determined by trypan blue staining. Results show cell growth as a percentage relative to NT cell numbers at day 5 and are mean ± s.e. mean, n = 3.

Incubation of either NaHS or GYY4137 in culture medium resulted in the release of detectable amounts of H_2_S as reflected by an increase in concentration of H_2_S (µM) following removal of aliquots and assay for methylene blue formation. Release of H_2_S from NaHS was rapid - peaking at or before 20 min and declining to undetectable levels by 90 min. In stark contrast, H_2_S release from GYY4137 was much lower (<10% of that observed with NaHS) but was sustained, remaining higher than baseline for up to 7 days. No release of H_2_S was apparent from ZYJ1122, a control for GYY4137 lacking sulfur and thus unable to form H_2_S, under the same experimental conditions for up to 7 days (Figure 1A). The chemical structures of GYY4137 and ZYJ1122 are shown in the inset to Figure 1A.

### Effect of NaHS and GYY4137 on cell growth and viability

The effect of NaHS, GYY4137 and ZYJ1122 on growth of three cancer cell lines i.e. MCF-7 (breast adenocarcinoma), MV4–11 (acute promyelocytic leukemia) and HL-60 (myelomonocytic leukemia), was monitored for 5 days. At each indicated interval, the number of live cells from each treatment group was recorded in triplicate. GYY4137 (400 µM) significantly reduced cell proliferation of all three cancer lines whereas both NaHS and ZYJ1122 were inactive (Figure 1B).

**Figure 2:**
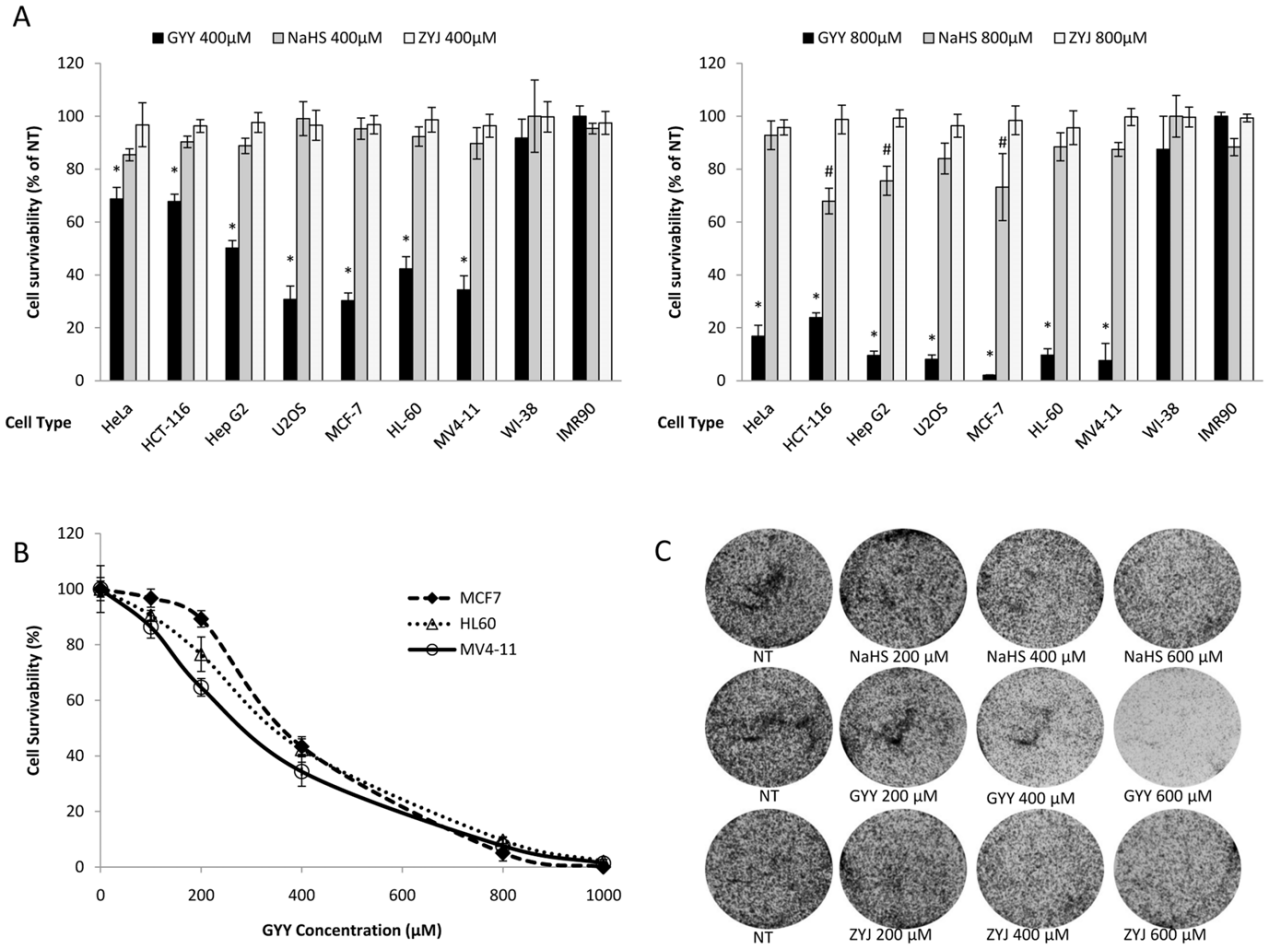
GYY4137 but not NaHS significantly affected cancer cell survivability. (A) The effect of treatment (5 days) of a range of cancer and non-cancer cells with NaHS, GYY4137 and ZYJ1122 (400 µM or 800 µM) as determined by trypan blue staining. Results show percentage of cell viability to non-treatment (NT) following incubation in the absence of drug treatment and are mean ± s.e. mean, n = 3, (^#^ *P*<0.05; *P<0.01). (B) Concentration-response curves showing the effect of GYY4137 treatment for 5 days on survival of MCF-7, HL-60 and MV4-11 cells. Results show mean ± s.e. mean, n = 3. (C) Representative photographs showing clonogenic survival assays of MCF-7 cells following exposure (5 days, 200–600 µM) to either NaHS (top row), GYY4137 (middle row) and ZYJ1122 (bottom row). NT =  non-treatment.

To determine the effect of two different concentrations (400 µM and 800 µM) of GYY4137, NaHS and ZYJ1122 on a wider panel of human cancer cell lines, cell survival of a further four cancer cell lines of different origins i.e. cervical carcinoma (HeLa), colorectal carcinoma (HCT-116), hepatocellular carcinoma (Hep G2) and osteosarcoma (U2OS) cells was determined in comparison with two normal human diploid fibroblasts (WI-38 and IMR90) (Figure 2A). Over a 5-day culture period, NaHS (400 µM) failed to influence the survival of any of the seven cancer lines tested. In contrast, the effect of GYY4137 on cell survival was much more profound with 30–70% (*P*<0.01) death in all cancer cell lines at the same concentration. A higher concentration of NaHS (800 µM) resulted in a further, albeit small, reduction in HCT-116, Hep G2 and MCF-7 cell survival (approximately 15–30%) although again no significant difference in cell survival was apparent in HeLa, HL-60, U2OS and MV4-11 cells. In contrast, GYY4137 at the same concentration, markedly reduced survival by approximately 75–95% in all cancer cell lines. The absolute degree of cell death caused by GYY4137 varied between cancer cell lines with greatest effect in HepG2, HL-60, MV4–11, MCF-7 and U2OS cells and least effect in HCT-116 and HeLa cells. For this reason, subsequent experiments were conducted using one or more of HL-60, MCF-7 and MV4-11 cancer cells. Importantly, neither NaHS nor GYY4137 significantly changed the survival of human non-cancer WI-38 and IMR90 fibroblasts. The sulfur-lacking control compound, ZYJ1122, was without significant effect on the survival of any cell line, suggesting that the observed effects of GYY4137 on cancer cells are likely due to H_2_S release. The concentration response relationship for GYY4137 (100–1000 µM) to reduce cell survival was also examined in MCF-7, HL-60 and MV4-11 cells. The IC_50_ values for this compound were 337.1±15.4, 389.3±16.8 and 341.8±21.2 µM (all n = 3**)** respectively (Figure 2B).

The effect of NaHS, GYY4137 and ZYJ1122 (200–600 µM) on survival of MCF-7 cells was also assessed *in vitro* using a clonogenic assay. Representative photographs are shown in Figure 2C. For these experiments, MCF-7 cells were plated in the presence or absence of drugs and cultured over a 10-day period. GYY4137 caused a concentration dependent loss of cell colony formation which was close to maximal at a concentration of 600 µM. Cell loss was not apparent in both NaHS and ZYJ1122 treated samples.

**Figure 3:**
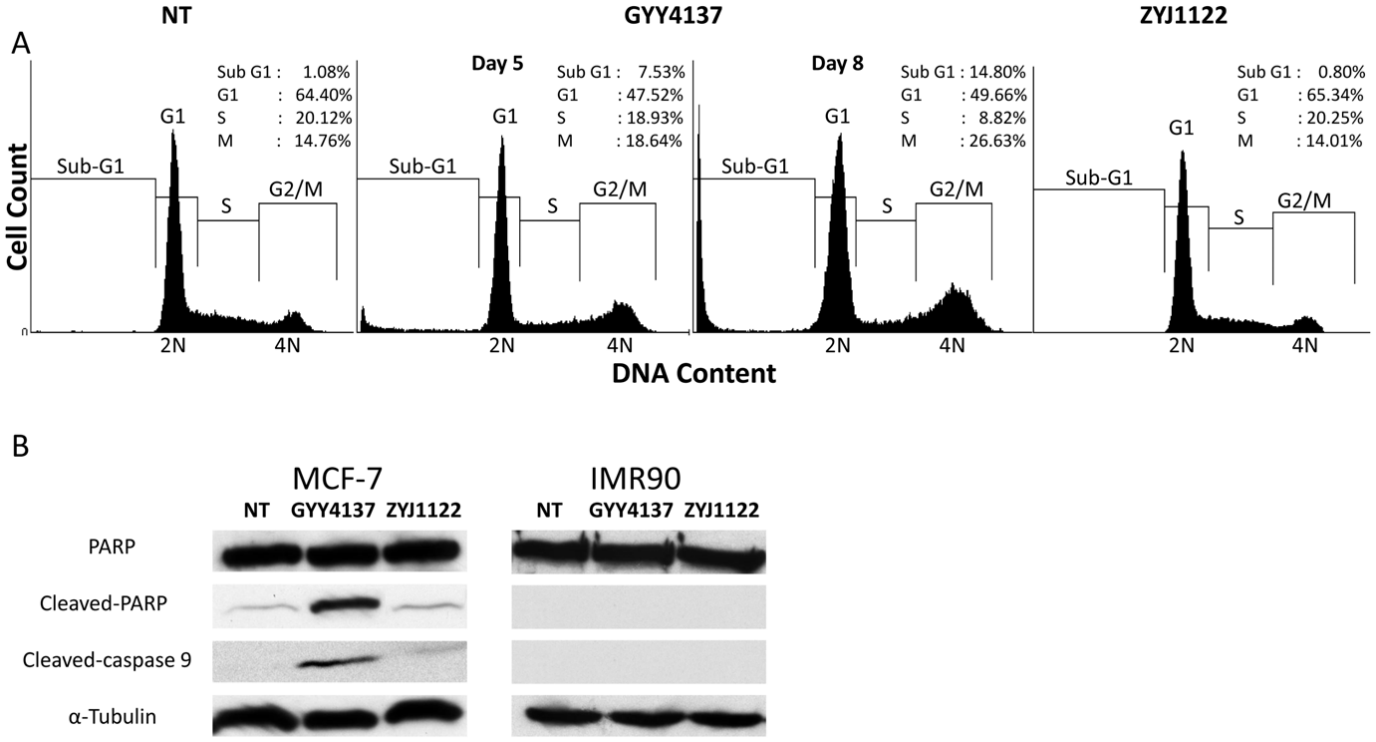
GYY4137 induced sub-G1 population and apoptosis in MCF-7. (A) Cell cycle analysis of MCF-7 cells after 5 days (NT and ZYJ1122) and 5 and 8 days (GYY4137) drug treatment. Insets show percentage distribution of cells in each cell cycle phase. Results shown are indicative of 3 individual experiments. NT: non-treatment. (B) Western blot analysis of apoptosis markers (α-cleaved-PARP, α-cleaved-caspase 9) of MCF-7 and IMR90 treated (5 days) with GYY4137 or ZYJ1122 (both 400 µM). The anti-cleaved caspase-9 antibody used detects the large fragment of caspase-9 following cleavage but does not recognize uncleaved procaspase-9. α-tubulin was used as a loading control. Results shown are indicative of 3 individual experiments.

The effect of GYY4137 (400 µM, 5 or 8 days) on MCF-7 cells was also examined using cell cycle analysis. The sub-G1 population of MCF-7 cells exposed to GYY4137 was significantly higher (*P*<0.05) compared either to non-treated cells or cells exposed to the same concentration of ZYJ1122 on day 5 (Figure 3A). Thus, the sub-G1 population of cells treated with GYY4137 represented 7.5% of the total cell population at day 5 and 14.8% at day 8 of treatment compared with approximately 1% of cells which either did not receive treatment or were exposed to ZYJ1122 (Figure 3A). In addition, there was a significant accumulation of 4N-DNA cell population in cells treated with GYY4137 (to 18.6% and 26.6% after 5 and 8 days of incubation respectively) as compared to either untreated (14.8%) or ZYJ1122-treated (14%) cells (Figure 3A). In further experiments, the possibility that GYY4127 triggers cancer cell death by promoting apoptosis was also studied. A strong signal for cleaved-PARP and activated caspase 9 was detected in MCF-7 samples treated with GYY4137 (400 µM, 5 days) with a greatly reduced signal in cells treated with ZYJ1122 (Figure 3B). Interestingly, no cleavage of PARP and no activation of caspase 9 were observed in IMR90 cells incubated with either GYY4137 or ZYJ1122.

### Effect of GYY4137 on tumor growth *in vivo*

**Figure 4:**
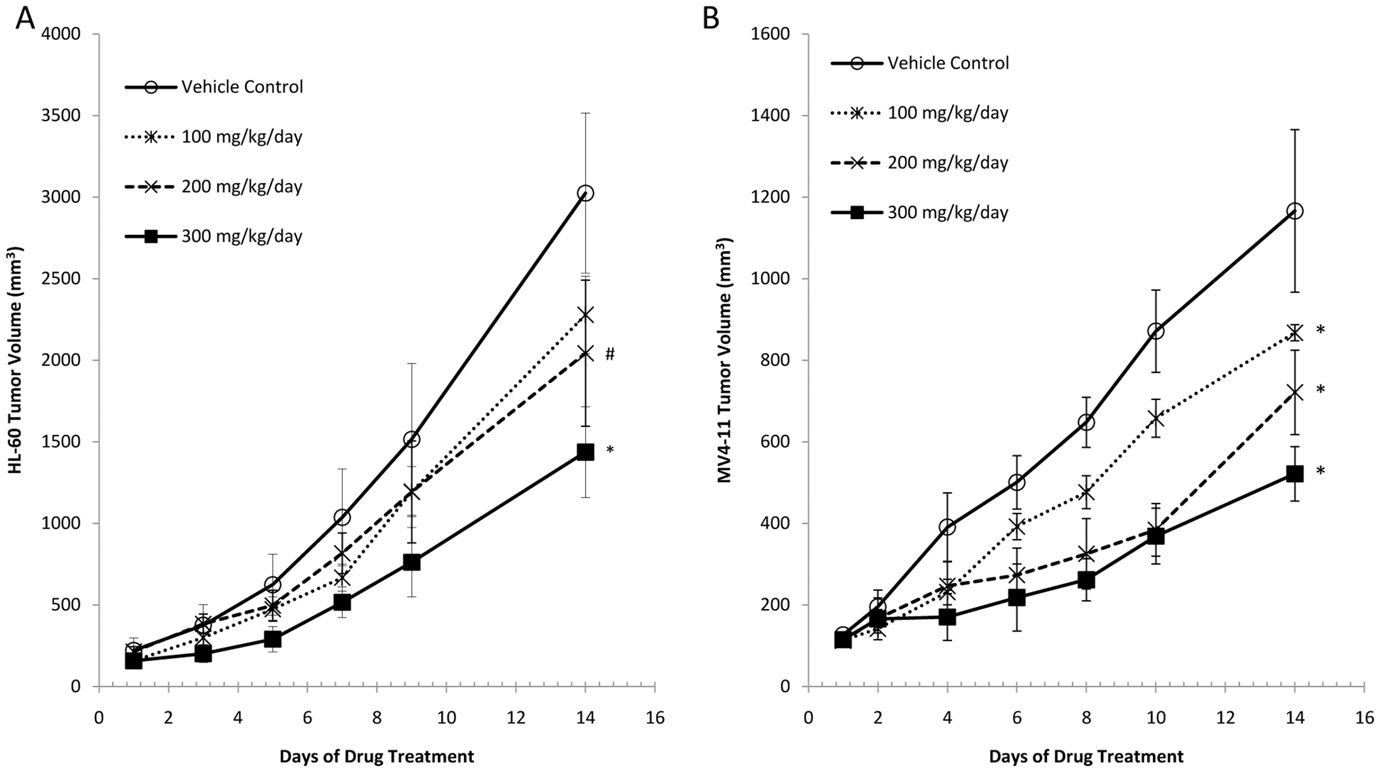
Effect of GYY4137 on tumor growth *in vivo*. Changes in volume of established tumors in, (A) HL-60 xenograft mice (B) MV4–11 xenograft mice treated daily with either GYY4137 (100, 200 and 300 mg/kg, i.p.) or vehicle control. Treatment with GYY4137 significantly reduced the tumor volume in both animal models, in a dose-dependent manner. Results show changes of tumor volume in mean ± s.e. mean (n = 4–6, # *P*<0.05; * *P*<0.01).

Subcutaneous transplantation of either HL-60 or MV4–11 cells resulted in time-dependent tumor growth in the SCID mouse (Figure 4A, B). Tumor volume at the end of the experiment was 3024±220 mm^3^ and 1166±199 mm^3^ (n = 4–6) in animals receiving daily vehicle injection and administered HL-60 and MV4-11 cells respectively. Administration of GYY4137 on a daily basis resulted in a significant (*P*<0.05) dose related inhibition of tumor growth in both sets of animals. GYY4137 (at the highest dose used i.e. 300 mg/kg) administered daily for 14 days reduced tumor volume by 52.5±9.2% (n = 6) and 55.3±5.7% (n = 4) in HL-60 and MV4–11 injected animals. Although not measured objectively in these experiments, GYY4137 treatment did not affect animal weight or gross behavior.

## Discussion

We report here that, (i) GYY4137 (but not NaHS) causes a concentration-dependent reduction in cancer cell survival, (ii) neither GYY4137 nor NaHS, using identical concentrations and experimental conditions, affected the survival of normal i.e. non-cancer cells, (iii) GYY4137 promoted cancer cell (MCF-7) but not normal cell (IMR90) apoptosis as indicated by measurement of sub-G1 population and by observation of cleaved PARP and cleaved caspase 9 and triggered cell cycle arrest of MCF-7 cells in the G_2_/M phase, (iv) the H_2_S concentration detected in medium containing MCF-7 cells exceeded ‘basal’ levels for up to 7 days after exposure to GYY4137 but for less than 2 h after exposure to NaHS, (v) ZYJ1122, a control for GYY4137 lacking sulfur and thus unable to form H_2_S, was inactive in all cases and, (vi) GYY4137 administered daily to immunodeficient mice for 14 days caused a dose-dependent reduction in tumor growth elicited by prior injection of one of two human leukemia cell lines.

Thus, the present data reveals, for the first time, an anti-cancer effect of the slow-releasing H_2_S donor, GYY4137. All cancer cells tested were susceptible to this compound albeit to different extents. It is now well established that NaHS releases large amounts of H_2_S over a short time period. In the present experiments, we show that GYY4137, like NaHS, also releases H_2_S following incubation in culture medium containing MCF-7 cells thereby confirming our previous observation of spontaneous H_2_S generation in aqueous media [8]. Since ZYJ1122 exhibited no anti-cancer activity in any of the *in vitro* models we conclude that the anti-cancer activity of both GYY4137 and NaHS (at high concentration) is very likely to be H_2_S-dependent. Perhaps surprisingly, GYY4137 exhibited greater cancer cell killing activity than did NaHS *in vitro* even though it leads to markedly lower concentrations of H_2_S in the cell medium. Thus, optimal killing of cancer cells by H_2_S under these experimental conditions would appear to occur at low concentrations of the gas spread over a period of several days as opposed to a much higher concentration achieved over a shorter time frame following exposure of cells to NaHS. It should be noted that whilst relatively high concentrations of GYY4137 (i.e. 400–800 µM) are required for this effect, the effective concentration of H_2_S generated is much less i.e. <20 µM based on the measurements in culture medium. However, we cannot rule out the possibility that GYY4137 may accumulate inside cancer cells and thereby release larger amounts of H_2_S intracellularly. With this proviso in mind, the present data implies that the rate at which cells are exposed to H_2_S as well as the concentration of H_2_S encountered is critical in determining the ability of this gas to promote cell killing.

Interestingly, neither GYY4137 nor NaHS caused significant killing of normal i.e. non-cancer cells suggesting that the effect of both H_2_S donors is specific for cancer cells. The mechanism of action of the cancer cell killing effect of GYY4137 has also been investigated. Treatment of MCF-7 cells with GYY4137 resulted in cell cycle arrest in the G_2_/M phase and promotion of apoptosis as evidence by increased sub-G1 population as well as the presence of both cleaved PARP and cleaved caspase 9. No significant effect either on cell cycle or on apoptosis was apparent in ZYJ1122-treated cells again suggesting that both of these effects were secondary to the sustained exposure of cells to low levels of H_2_S. The ability of H_2_S to cause cancer cell killing in this way has not previously been reported. However, H_2_S has previously been shown to affect both cell cycle and apoptosis. For example, H_2_S promotes cell cycle entry and proliferation of intestinal IEC-18 cell *in vitro* by activating MAPK [13]. H_2_S can also exhibit both pro- (e.g. [14]) and anti- (e.g. [15]) apoptotic activity depending on the cell type studied and the experimental conditions, particularly the concentration of H_2_S used. A number of potential molecular targets have been implicated in the effect of H_2_S on apoptosis including p38 and caspase-3 [15], [16], other MAPK such as MEK and JNK [17] as well as augmented production of heat shock protein (HSP-90) [18]. Whilst the precise cellular mechanism of action of GYY4137 remains unclear, it seems not unreasonable that H_2_S, released slowly over a period of several days, may affect redox mechanisms within the cell to bring about the anti-cancer effect. In this light, it might be of interest to assess the effect of GYY4137 on reactive oxygen species generation and anti-oxidant enzyme levels in cancer cells.

In conclusion, GYY4137 exhibits anti-cancer cell activity both *in vitro* and *in vitro*. We propose that GYY4137 breaks down slowly to yield H_2_S which, by a combination of cell cycle arrest and promoting apoptosis, inhibits tumor growth. No cell death was apparent in non-cancer cells. Whether such cells simply break down H_2_S at a faster rate or whether cancer cells are uniquely sensitive to the killing effect of this gas requires further study. The finding that cancer cells can be killed selectively when exposed to relatively small amounts of H_2_S over a relatively long time period is the key. This observation needs to be borne in mind in any future work examining the part played by H_2_S in cancer cell survival and also in the development of novel H_2_S-based anti-tumor agents.

## Supporting Information

Figure S1 **^1^H NMR spectrum of GYY 4137.**
(TIF)

Figure S2 **Mass spectrometry spectrum of GYY 4137.**
(TIF)

Figure S3 **^1^H NMR spectrum of ZYJ1122.**
(TIF)

Figure S4 **Mass spectrometry spectrum of ZYJ1122.**
(TIF)
