# Supplementary figures and images for: The Slow-Releasing Hydrogen Sulfide Donor, GYY4137, Exhibits Novel Anti-Cancer Effects In Vitro and In Vivo
Source: PLoS One. 2011 Jun 20;6(6):e21077. doi: 10.1371/journal.pone.0021077 (PMC3119065; doi:10.1371/journal.pone.0021077)

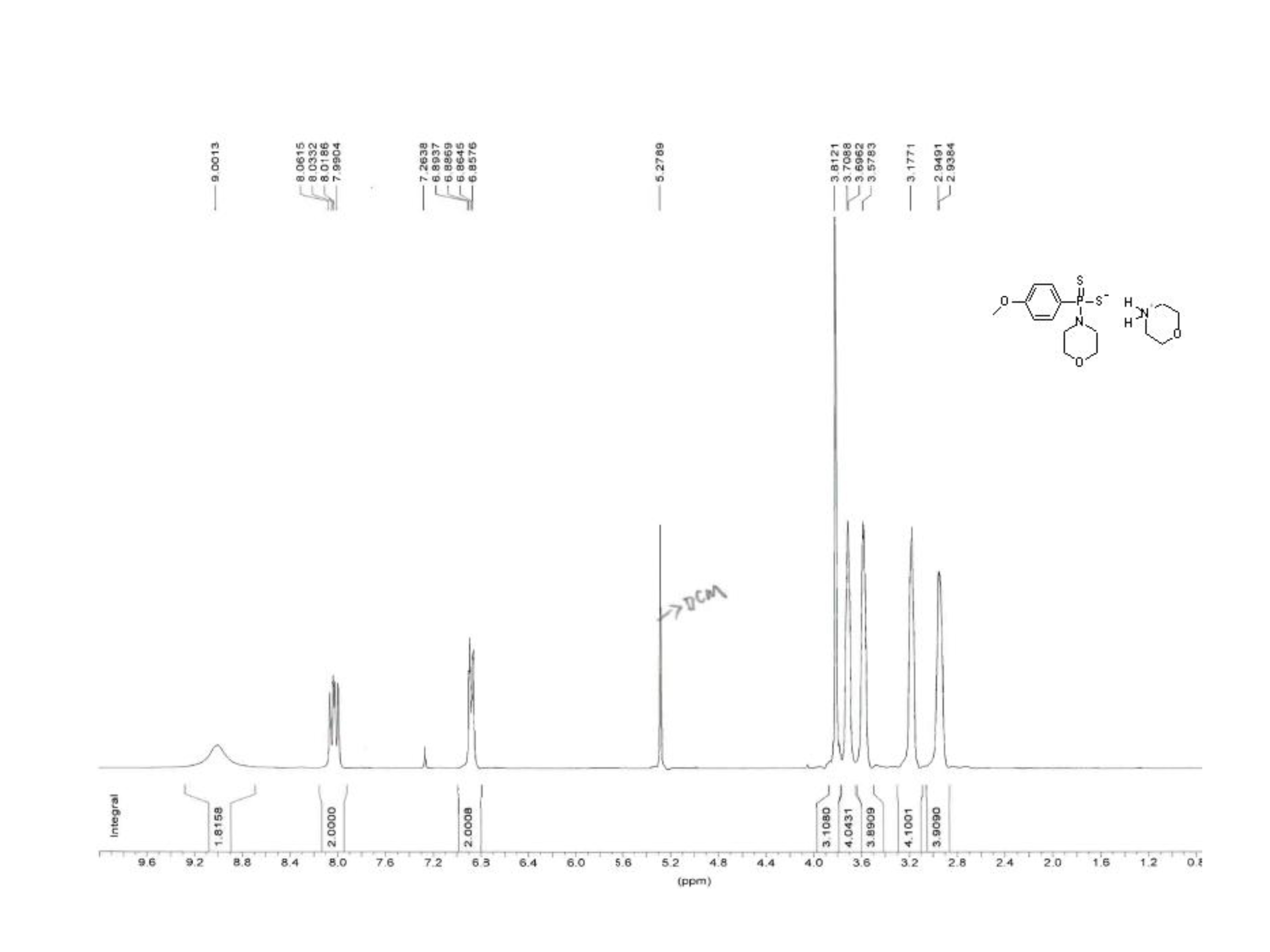

Supplement: Figure S1 — 1H NMR spectrum of GYY 4137. (TIF) [file pone.0021077.s001.tif]

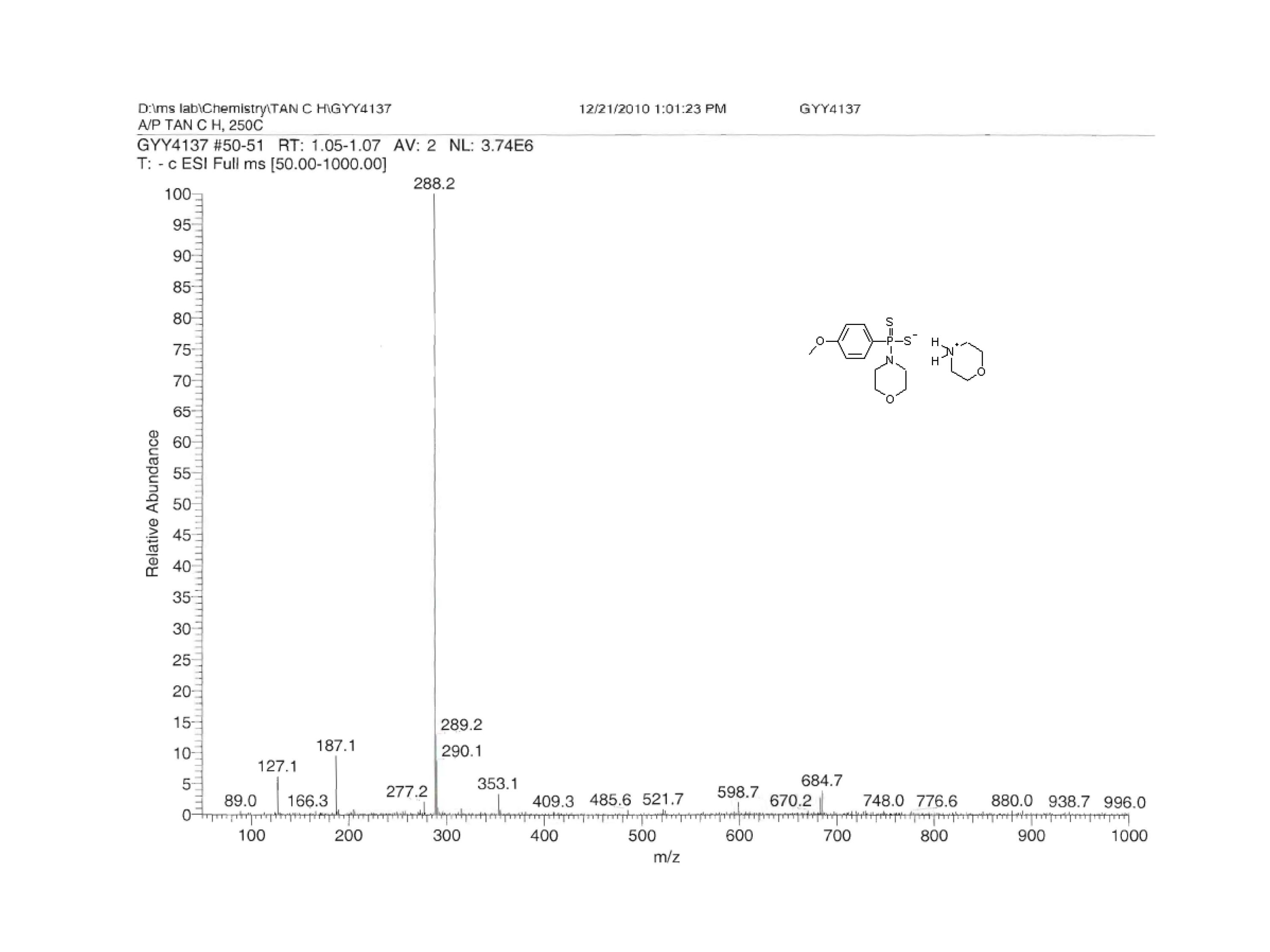

Supplement: Figure S2 — Mass spectrometry spectrum of GYY 4137. (TIF) [file pone.0021077.s002.tif]

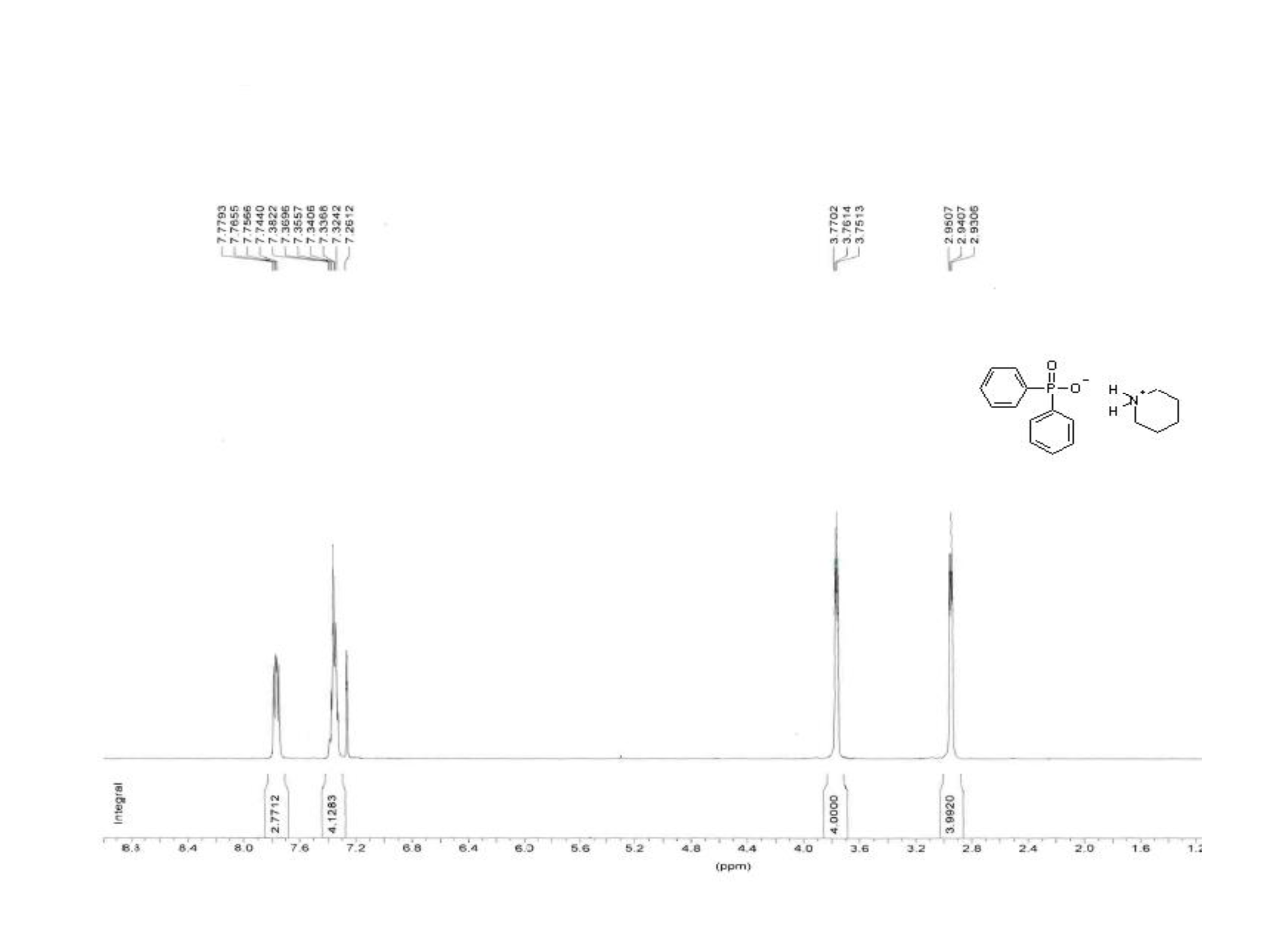

Supplement: Figure S3 — 1H NMR spectrum of ZYJ1122. (TIF) [file pone.0021077.s003.tif]

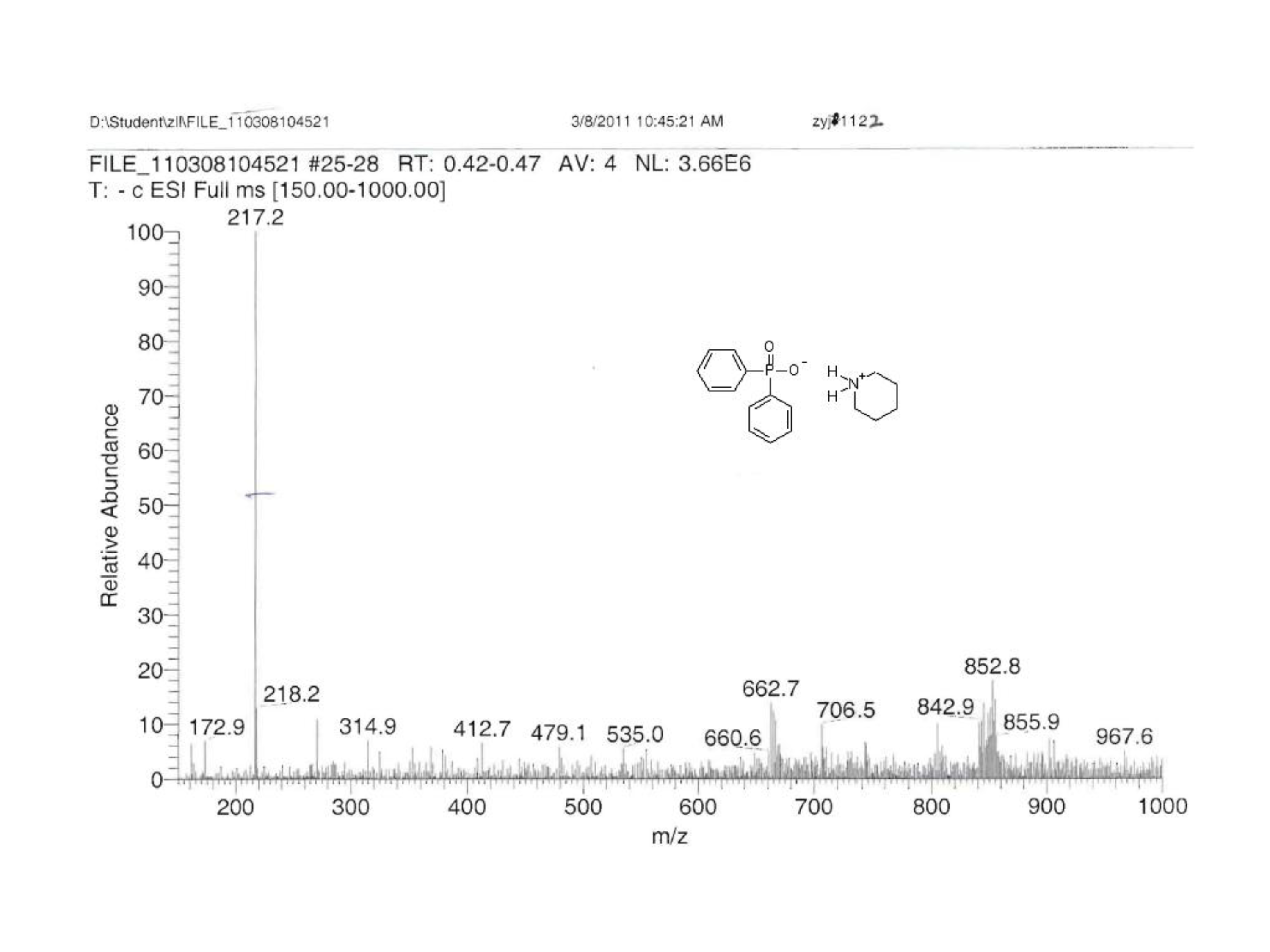

Supplement: Figure S4 — Mass spectrometry spectrum of ZYJ1122. (TIF) [file pone.0021077.s004.tif]
